# Comment on MaaniHessari, N.; van Schalkwyk, M.C.; Thomas, S.; Petticrew, M. Alcohol Industry CSR Organisations: What Can Their Twitter Activity Tell Us about Their Independence and Their Priorities? A Comparative Analysis. *Int. J. Environ. Res. Public Health* 2019, *16*, 892

**DOI:** 10.3390/ijerph16132421

**Published:** 2019-07-08

**Authors:** Fiona Sim, Jonathan Chick, Stephen Neidle, Graham Ogden, Sarah Jarvis, Iona Lidington, Helene Leslie

**Affiliations:** Independent Medical Advisory Panel to the Drinkaware Trust, Salisbury House, London EC2M 5SQ, UK

We noted with concern that you published recently (12 March 2019) an article about so-called “Alcohol industry CSR Organisations”, which selectively targets criticism of Drinkaware. Drinkaware is a well-established independent charity which has a Memorandum of Understanding with the UK Government confirming its purpose of public education.

As its independent medical and scientific advisors, we are disturbed at the objectives of this study, which are intended to undermine the charity’s independence and value. Describing an alcohol education charity as an alcohol industry CSR organisation is, at best inaccurate, and in reality, highly misleading. To imply that “the purpose of such bodies is the protection of the alcohol market, and of the alcohol industry’s reputation” is not based on any factual evidence, but on the beliefs of the authors: as its independent advisors, we are confident that there is no such purpose. Indeed, Drinkaware’s ability to reach millions of lay people to educate them on the harms of alcohol is well recognised and its record in using Twitter effectively for this purpose, is unrivalled. We would challenge anyone to explain how encouraging people to drink less could be interpreted as “the protection of the alcohol market”. The charity’s website [www.drinkaware.co.uk] is both a testament to its purpose and a contribution to improving people’s health, and is transparent about its funding. Its encouragement of drink-free days parallels that of Cancer Research UK, hardly an organisation that protects the alcohol market [1].

Following publication of another article on this subject in 2017 by the same corresponding author [2] we submitted a Letter to the Editor of the journal concerned to point out the lack of evidence for the assertions made: this Letter was published [3]. We note, however, that although the present article relies heavily on that earlier article, no attempt has been made to offer a balanced perspective, nor to acknowledge published rejection of many of the unsupported assertions in that article. Given the critical stance of the paper, we wondered if perhaps the journal had encountered difficulty in identifying external reviewers who might have been alert to possible bias? It is very concerning that no reviewer appears to have spotted that the only “validation” of the authors’ assertions, is to their own previous paper.

We wish to express our serious concern at the failings that have led to the unfounded assertions made in the earlier article being the explicit basis of another article, which persists in making such unwarranted assertions that do not stand up to scientific scrutiny.
